# Identification of novel *CDH23* heterozygous variants causing autosomal recessive nonsyndromic hearing loss

**DOI:** 10.1007/s13258-024-01611-w

**Published:** 2025-01-08

**Authors:** Baoqiong Liao, Wuming Xie, Rutian Liu, Qi Zhang, Ting Xie, Dan Jia, Shuwen He, Hailong Huang

**Affiliations:** 1 Ganzhou Maternal and Child Health Hospital, Ganzhou, Jiangxi China; 2 Medical Genetic Diagnosis and Therapy Center of Fujian Maternity and Child Health Hospital College of Clinical Medicine for Obstetrics & Gynecology and Pediatrics, Fujian Medical University, Fujian Provincial Key Laboratory of Prenatal Diagnosis and Birth Defect, Fuzhou, Fujian China; 3 Ganzhou People’s Hospital, Ganzhou, Jiangxi China; 4 Department of Chemistry and Molecular Biology, Gothenburg University, Gothenburg, Sweden

**Keywords:** Nonsyndromic hearing loss, *CDH23*, Autosomal recessive, Whole-exome sequencing, Noncanonical splice site variant

## Abstract

**Background:**

Hearing loss adversely impacts language development, acquisition, and the social and cognitive maturation of affected children. The hearing loss etiology mainly includes genetic factors and environmental factors, of which the former account for about 50–60%.

**Objective:**

This study aimed to investigate the genetic basis of autosomal recessive non-syndromic hearing loss (NSHL) by identifying and characterizing novel variants in the *CDH23* gene. Furthermore, it seeks to determine the pathogenic potential of the noncanonical splice site variant c.2398-6G > A.

**Methods:**

Comprehensive clinical evaluation and whole-exome sequencing (WES) were performed on the girl. The WES analysis revealed two novel variants in the *CDH23* gene, associated with nonsyndromic deafness 12 (DFNB12). To further explore the pathogenicity of these variants, functional studies involving in vivo splicing analysis were performed on the novel noncanonical splice site variant, c.2398-6G > A, which was initially classified as a variant of uncertain significance (VUS).

**Results:**

Whole-exome sequencing of the patient identified two compound heterozygous variants in *CDH23*: c.2398-6G > A, a noncanonical splice site variant, and c.6068C > A (p. Ser2023Ter), a nonsense mutation. In vitro splicing assays demonstrated that c.2398-6G > A caused aberrant splicing, leading to a frameshift (p. Val800Alafs*6) and the production of a truncated protein, as confirmed by structural protein analysis. The study revealed novel mutations as likely pathogenic, linking both variants to autosomal recessive NSHL.

**Conclusions:**

Our analyses revealed novel compound heterozygous mutations in *CDH23* associated with autosomal recessive NSHL, thereby expanding the mutational landscape of *CDH23*-related hearing loss and increasing knowledge about the *CDH23* splice site variants.

## Introduction

Hearing loss is one of the most common sensory deficit diseases, which can occur in people of all ages, leading to severe speech communication disorders and affecting the patients, quality of life. Deafness exhibits a high prevalence, with a complex and multifactorial etiology. According to the World Hearing Report released by the WHO in 2021, there are as many as 1.5 billion people with deafness in the world at present, and it is expected to rise to 2.5 billion by 2050 (Chadha et al. 2021). The incidence of deafness in infants worldwide is about 2–6% (Olusanya 2012).

The hearing loss etiology mainly includes genetic factors and environmental factors, of which the former account for about 50–60% (Yuan et al. 2020). Congenital hearing loss stems from genetic causes that are split into two categories: syndromic, involving other medical conditions, and non-syndromic, where hearing loss is the sole manifestation. Non-syndromic hearing loss (NSHL) constitutes 75% of all inherited hearing impairments. Of these cases, about 80% are caused by autosomal recessive genes, 15–20% by autosomal dominant genes, and 1–2% by either mitochondrial or X-linked mutations (Alford et al. 2014). Syndromic hearing loss is related to other symptoms, comprises the remaining 30% of genetic hearing loss cases (Smith et al. 2005; Lammens et al. 2013). The rapid development of genetic testing technology, especially the next-generation sequencing, has promoted the exploration of the pathogenesis of hereditary deafness. A growing number of deafness genes have been identified, has been clear as many as 224 kinds of deafness genes. The common pathogenic factors in China are *GJB2, SLC26A4*, and *CDH23* (Du et al. 2016).

Recessive mutations of the *CDH23* gene (OMIM: 605,516) are responsible for both nonsyndromic deafness 12 (DFNB12, OMIM: 601,386) and Usher syndrome type 1D (USH1D, OMIM: 601,067) (Bolz et al. 2001; Bork et al. 2001a; Astuto et al. 2002). DFNB12 is characterized by prelingual-onset sensorineural NSHL, without the impairment of visual or vestibular functions. In contrast, individuals with USH1D exhibit severe phenotype, encompassing congenital severe to profound deafness, variable vestibular areflexia, and progressive adolescent-onset vision loss due to retinitis pigmentosa (RP) (Bork et al. 2002; Oshima et al. 2008; Friedman et al. 2011). The encoded protein cadherin 23 (*CDH23*) is a member of the cadherin super family which constitutes calcium-dependent cell–cell adhesion glycoproteins and is known to be expressed in both the inner and outer hair cells in the cochlea. Encoded protein cadherin 23 comprises the “Tip Link” structure of the stereocilia important for hair cell function. *CDH23* has essential roles in establishing and maintaining the proper organization of the stereocilia bundle of hair cells in the cochlea and vestibule during late embryonic and early postnatal development (Ramzan et al. 2020). Notably, a number of *CDH23* mutations have been described to date, revealing an interesting genotype–phenotype correlation shown in Table 1.

**Table 1:** Splicing mutations described in the CDH23 gene, with their nucleotide position and associated phenotype (from the Human Gene Mutation Database)

Mutation	HGMD access ID	Mutation type	Disease
2177-2A > G	CS1110166	IVS19 as A-G -2	Usher syndrome 1
2176+1G > C	CS1610394	IVS19 ds G-C + 1	Deafness, non-syndromic, autosomal recessive
2290-1G > A	CS2329085	IVS20 as G-A -1	Hearing loss, non-syndromic
2289+1G > A	CS021740	IVS20 ds G-A + 1	Usher syndrome 1
2289+6T > G	CS186197	IVS20 ds T-G + 6	Hearing loss, non-syndromic
2398-1G > T	CS1613921	IVS21 as G-T -1	Retinal disease
2587+1G > T	CS088228	IVS22 ds G-T + 1	Usher syndrome 1
6030T > G	CM2257689	Phe2010Leu	Hereditary diffuse gastric cancer
6049G > A	CM077923	Gly2017Ser	Usher syndrome 1
6083A > C	CM190105	Asp2028Ala	Hearing loss, non-syndromic
6085C > T	CM074086	Arg2029Trp	Hearing loss, non-syndromic
6098C > A	CM226757	Ser2033Term	Hearing loss
6133G > A	CM010181	Asp2045Asn	Non-syndromic autosomal recessive deafness

DFNB12 is characterized by prelingual-onset sensorineural hearing loss (SNHL) with no vestibular or visual involvement, typically showing a stable, non-progressive course (Zhong 2013). In contrast, USH1D, a more severe form, is associated with congenital profound hearing loss, vestibular areflexia, and progressive vision loss due to retinitis pigmentosa (RP), which begins in adolescence (Tb et al. 2011). This spectrum of phenotypes is largely influenced by the specific mutations in the CDH23 gene, which exhibit a genotype–phenotype correlation that can provide valuable insights into diagnosis and prognosis. In particular, compound heterozygous mutations in CDH23 have been increasingly recognized as an important cause of DFNB12 and USH1D, and their functional consequences have been studied extensively in recent years (Bork et al. 2001b). Advances in molecular genetic testing and the ability to detect such mutations using techniques such as whole-exome sequencing (WES) and Sanger sequencing have enabled more accurate diagnosis and better clinical management of affected families.

In the current study, we describe a Chinese family in which the youngest daughter had profound high-frequency progressive NSHL, without any vestibular or ocular involvement. Detailed clinical and molecular genetic analyses were performed through WES and Sanger sequencing. We identified two novel candidate variants in *CDH23*, one of which is intronic variation in the junctional region (c.2398-6G > A), was evaluated “variant of uncertain significance (VUS)” based on the latest ACMG guidelines (Richards et al. 2015). To further evaluate this novel VUS variant, we performed in vivo splicing analysis. We confirmed the compound heterozygosis in *CDH23* as the cause of NSHL in the girl with late onset autosomal recessive hearing loss. The implications of our discovery are valuable in the clinical diagnosis, prognosis, and treatment of patients with *CDH23* pathogenic variants.

## Material and methods

### Patients and clinical data

An 8-year-old Chinese girl presented with hearing loss after suspicion based on regression of speech. She is the second child born to healthy, non-consanguineous parents and has a healthy elder sister. No significant medical history was noted during the prenatal, delivery, and postpartum periods. The patient successfully passed the newborn hearing screening, with no abnormalities detected at that time. Her early language milestones were within the expected range. She began babbling at 8–9 months and spoke her first words by 12 months. By the age of 2 years, she was capable of speaking longer words. However, a gradual regression in her language skills was observed starting at the age of 3 years. Currently, her verbal communication is predominantly limited to the use of repetitive and simple words. She is using a hearing aid, which provides some improvement in her auditory capabilities.

### Clinical evaluation of subject

The girl was thoroughly examined in the department of paediatrics and otolaryngology at Ganzhou Maternal and Child Health Hospital. Detailed medical histories and physical examinations, such as computerized tomography (CT) scans of the temporal bonehead, magnetic resonance (MR) labyrinthography and eye fundus were carried out to exclude any possible environmental causes or syndromic forms of hearing loss. Audiological evaluation included tympanometry, distortion product otoacoustic emission (DPOAE), click-evoked air-conduction auditory brain-stem response (AC-ABR), click-evoked bone-conduction auditory brain-stem response (BC-ABR), pure-tone audiometry (PTA). The hearing levels were determined by the thresholds of AC-ABR, NB CE-chirp ASSR, or PTA. Further, according to the World Report on Hearing in 2021, the grades of hearing loss were classified as mild (20 to < 35 dB HL), moderate (35 to < 50 dB HL), moderately severe (50 to < 65 dB HL), severe (65 to < 80 dB HL), or profound (80 to < 95 dB HL), complete or total (> 95 dB HL) by the average threshold of PTA air conduction in 500 Hz, 1000 Hz, 2000 Hz and 4000 Hz. In order to estimate the progress of her hearing levels, we evaluated her hearing in her 7 and 7.5 years old respectively by DPOAE, AC-ABR and BC-ABR. PTA was conducted when she was 8 years old.

### Sample collection

This research was approved by the Ethics Committee of the Ganzhou Maternal and Child Health Hospital. Peripheral venous blood samples were taken from the girl, her parents and her elder sister following the acquisition of informed consent from all participants. RNA was extracted using the PAXgene Blood RNA Kit (PreAnalytiX, Hombrechtikon, Switzerland). In addition, DNA was extracted from the peripheral blood using the QIAamp DNA Mini Kit for blood (Qiagen, Hilden, Germany). Trio-based WES was performed by KingMed Diagnostics (Guangzhou, China), including exome library preparation, sequencing, and data analysis. Variant screening was based on clinical phenotypes of the affected subjects, population database (dbSNP, 1,000 Genome, ExAC), disease database (OMIM, HGMD, Clinvar), and biological information prediction tools (SIFT, Polyphen2, Mutation Taster and Splice AI). Two novel variants of *CDH23* were identified in the girl, one is a splicing variant (*CDH23*: c.2398-6G > A) inherited her mother, and the another is a nonsense variant (*CDH23*: c.6068C > A), inherited from her father. The two *CDH23* variants were confirmed by Sanger sequencing. To identify the potential impact of the c.2398-6G > A noncanonical splice variant of *CDH23*, the RDDCSC online in silico splice site prediction software (https://rddc.tsinghua-gd.org/search-middle?to=SplitToolModel, accessed on 23 April 2024) was used.

### In vivo splicing analysis of CDH23 transcripts in the patient’s mother

Following prior confirmation of CDH23 expression in blood, samples from the patient’s mother and a control were utilized to analyze the splicing pattern of exons 22, 23, and 24 via RT-PCR. Their blood was collected in PAXgene Blood RNA tubes, and RNA was extracted using PAXgene Blood RNA Kit (PreAnalytiX, Hombrechtikon, Switzerland). Two rounds of PCR were performed using nested primers: the first PCR was performed using Complementary DNA (a total of two sets of DNA) as a template, with *CDH23*-1F (5-GAAATCACCACCACGTCTCT-3) and *CDH23*-1R (5-TGCTGCTGTTGATGAGGAAG-3) as primers for 30 cycles. The second PCR was performed using products from the first round of PCR as a template, with *CDH23*-2F (5-GTGGGCCACAACCAGAAAAC-3) and *CDH23*-2R (5-TTCAAGCACCTCGGCCACAA-3) as primers for 30 cycles.

### Protein structure prediction

The protein sequence with 3354 amino acid residues of *CDH*23 was downloaded from uniprot web (https://www.uniprot.org/). The wild-type, mutant-type1 (*CDH*23:c.6068C > A) and mutant-type2 (*CDH23*:c.2398-6G > A) 3D structure of the *CDH*23 protein were predicted using AlphaFold web server (https://golgi.sandbox.google.com/about) (Abramson et al. 2024). The best model was selected based on pLDDTs prediction scores. The higher the score, the more confident is the structure. Prediction models editing was performed was visualized using PyMOL program (https://pymol.org/).

### Bioinformatics analysis

The protein–protein interaction (PPI) network associated with *CDH23* was constructed using STRING (https://string-db.org/). The amino acid sequence of the *CDH23* protein, comprising 3354 residues, was retrieved from the UniProt database (https://www.uniprot.org/). This sequence was used as the input query for STRING, where the parameters were set to ensure high-confidence interactions were captured. The confidence score threshold was adjusted to include only interactions supported by strong evidence such as experimental data, co-expression, and co-occurrence analyses.

## Results

### Clinical description and audiological features

Given the association of various *CDH23* mutations with usher syndrome, the patient underwent a thorough examination to rule out related phenotypes. For example, fundus examinations revealed no macular changes in both eyes. A comprehensive medical history was obtained using the previously mentioned questionnaire. Additionally, the patient did not exhibit other visual symptoms such as night blindness, visual field loss, or reduction in central vision. A CT of the temporal bone revealed normal pneumatization of both mastoids. The inner ear structures, including the cochleae, vestibules, semicircular canals, and middle ear structures, were all found to be normal. MR labyrinthography further confirmed that both the internal auditory canal and the labyrinth of the inner ear membrane were intact.

**Fig. 1:**
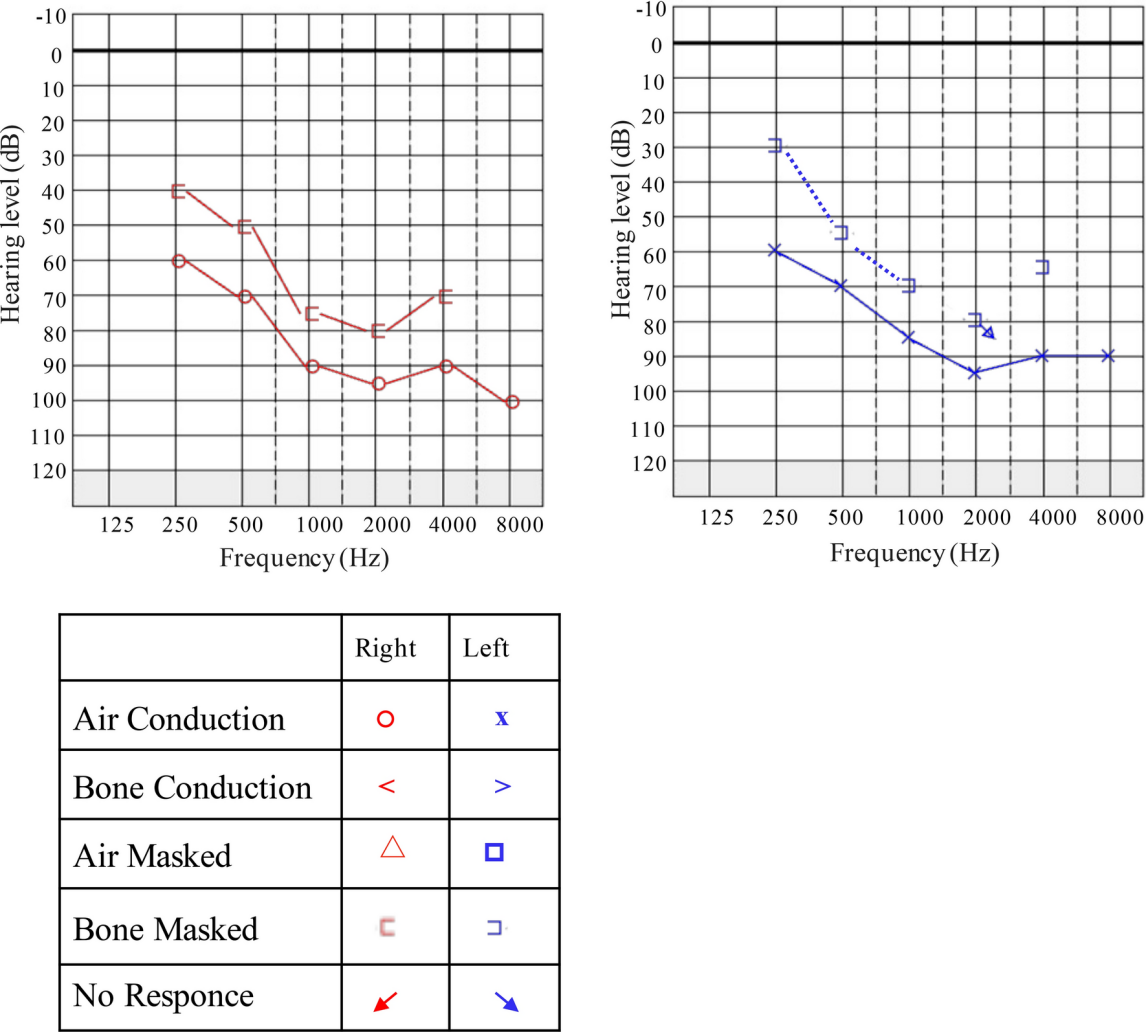
Pure Tone Audiometry (PTA) assessments reveal profound bilateral sensorineural hearing loss in the proband at 8 years of age. The audiometric data were graphically represented using Audiogram Creator (https://audiogramcreator.com) according to the original graphs. The legend symbol indicating various audiometric thresholds and conditions are shown above. The audiogram demonstrates the progression of profound hearing loss across both ears at age 8, with an average threshold of 85 dB HL for air conduction, indicating severe hearing impairment

The patient exhibited a progressive nature of hearing loss, as confirmed by audiograms obtained at different ages. Audiological assessments, including tympanometry, DPOAE, AC-ABR and BC-ABR were conducted in her 7 and 7.5 years old, respectively (Table 2). ABR thresholds, which can predict pure-tone behavioral thresholds across a wide frequencies range (Gorga et al. 2006), revealed severe hearing loss in both ears at age 7. Six months later, the left ear hearing become worse up to profound hearing loss. What^’^s more, tympanometry at age 7.5 was showed flat curve at 1000 Hz in the left ear. By age 8, the average threshold of PTA air conduction was approximately 85 dB HL of two ears, indicating profound hearing loss (Fig. 1).

**Table 2:** The outcomes of tympanometry, DPOAE, click-evoked AC- and BC-ABRs in 7 and 7.5 years old of the proband

Age at test (year)	Ear	Tympanometry	DPOAE	AC-ABR 2000-4000 Hz (dBnHL)	AC-ABR 500 Hz (dBnHL)	AC-ABR 1000 HZ (dBnHL)	BC-ABR (dBnHL)
7	L	Positive peak	Refer	70	65	75	45
	R	Positive peak	Refer	70	65	75	45
7.5	L	Flat	Refer	80	85	90	45
	R	Positive peak	Refer	65	70	80	45

### WES and variant assessment

**Fig. 2:**
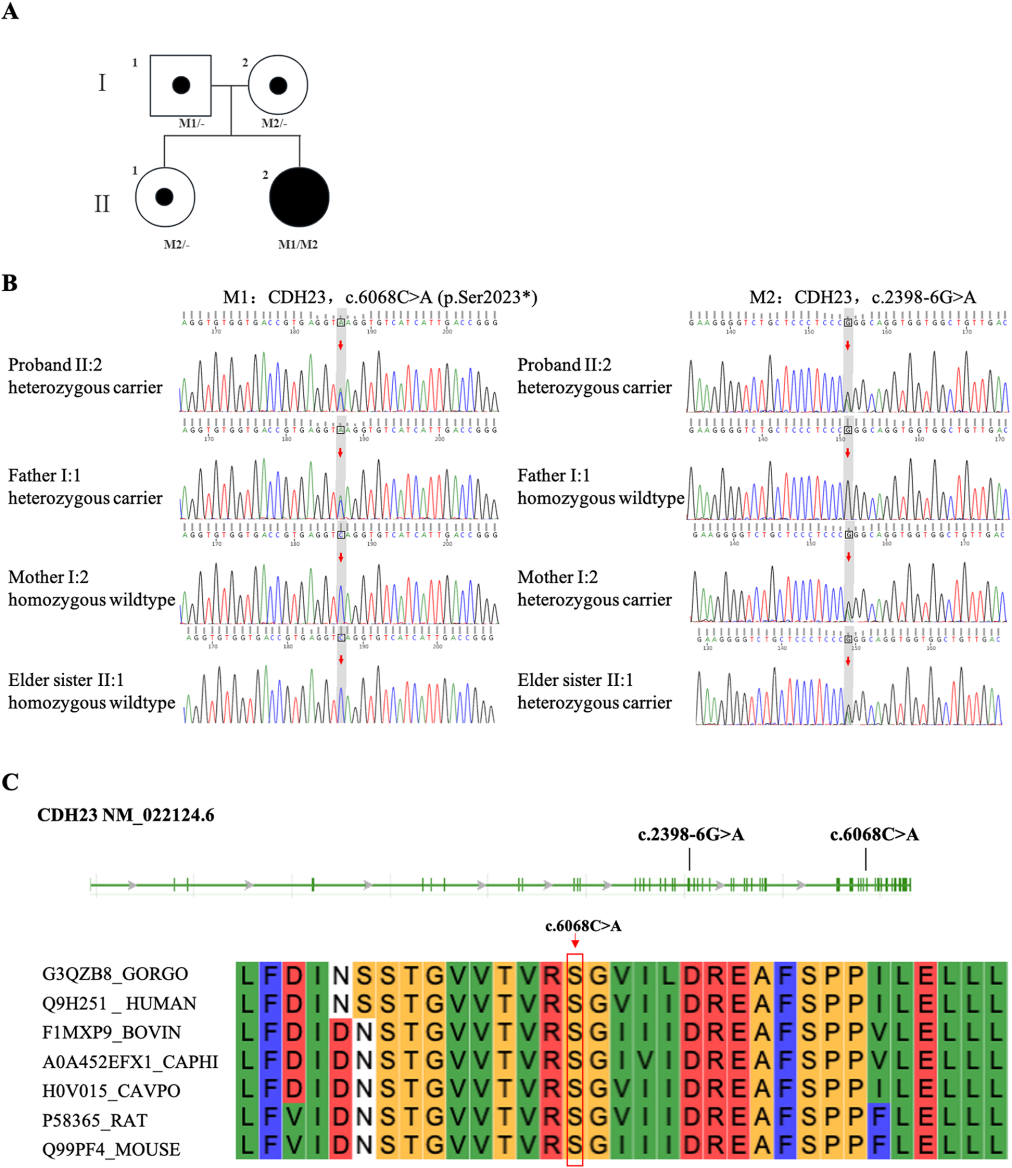
Composite illustration detailing the genetic analysis of the study family. **a** The pedigree chart illustrates the inheritance pattern of NSHL within the family. Circles and squares denote females and males, respectively. The arrow indicates the proband. Fully shaded symbols represent individuals diagnosed with deafness, while partially shaded symbols denote carriers of *CDH23* mutations. The genotypic data for the identified mutations in the *CDH23* gene are indicated below each tested family member. *CDH23*: M1/- or M2/- indicate heterozygous carriers of the c.6068C > A, (p. Ser2023*) and c.2398-6G > A, respectively. *CDH23*: M1/M2 indicates compound heterozygous individuals. **b** Sanger sequencing results for the c.6068C > A and c.2398-6G > A mutations in all family members. Red arrows highlight the specific nucleotide positions of these mutations. The results demonstrated that the compound heterozygous mutation cosegregated with the HL phenotype, consistent with the recessive inheritance observed in the pedigree. **c** Diagram of the location of *CDH23* mutations. The two identified mutations c.2398-6G > A and c.6068C > A are located in distinct regions of the gene. Key residues, particularly those at positions such as Ser2023, are shared across humans, gorillas, bovines, and rodents, suggesting their critical function in the protein’s structural integrity

Trio-based WES was performed, which an average depth of coverage of 138 reads was obtained with 98% of targeted regions covered at ≥ 20 ×, and three variants, c.2398-6G > A and c.6068C > A in *CDH23* (RefSeq NM_022124.6) and c.109G > A in GJB2 (RefSeq NM_004004.6), were considered as potential candidates. The pathogenic variants of *CDH23* and *GJB2* were related to the “hearing loss” and “regression of speech”. *CDH23* mutations in compound heterozygotes were detected in the proband: c.2398-6G > A and c.6068C > A, which were inherited from the mother and father, respectively. The elder sister inherited c.2398-6G > A variant from the mother. Pedigree of the family with NSHL was shown in Fig. 2a. Both variants were confirmed by Sanger sequencing (Fig. 2b). The c.6068C > A (p. Ser2023*) is a nonsense variant in *CDH23* that induces a premature stop codon in place of serine at position 2023. This serine residue is highly conserved across multiple species, including gorilla, human, bovine, goat, guinea pig, rat, and mouse, indicating its important role in maintaining the structural and functional integrity of the protein across different organisms (Fig. 2c). This variant lead to early termination of amino acid translation and may impact mRNA expression, which was not identified in population databases (1000 Genomes, Exome Aggregation Consortium, and Genome Aggregation Database). According to the American College of Medical Genetics and Genomics (ACMG) (Richards et al. 2015) guidelines for interpretation of sequence variants, we considered this variant to be likely pathogenic (PVS1 + PM2_Supporting). The c.2398-6G > A inherited her mother was present in GnomAD databases with extremely low minor allele frequency (MAF) (MAF = 6.574 × 10^−5^). In silico prediction indicated that the *CDH23* c.2398-6G > A variant may affect slicing through activating a new acceptor site or causing exon skipping (Table 3).

**Table 3:** The results of RDDCSC

Predicted signal	Predicted splicing map	Interpretation
New Acceptor site		Inserting 4 bp, premature termination,
Exon skipping		Deleting 190 bp, premature termination

Both splice patterns indicate the potential alteration of splicing in CDH23 c.2398-6G > A variant

Primitively, we considered this variant to be VUS (PM2 + PP3). The c.109G > A (p.Val37Ile) variants in GJB2 is classified as pathogenic for autosomal recessive nonsyndromic hearing loss, exhibiting variable expressivity and incomplete penetrance, according to the ClinGen Hearing Loss Expert Panel (Shen et al. 2019). The variants may lead to mild or moderate HI with reduced penetrance. Homozygous c.109G > A variants in GJB2 were detected in each member of this family including proband^’^s normal parents and sister.

### Splicing analysis

**Fig. 3:**
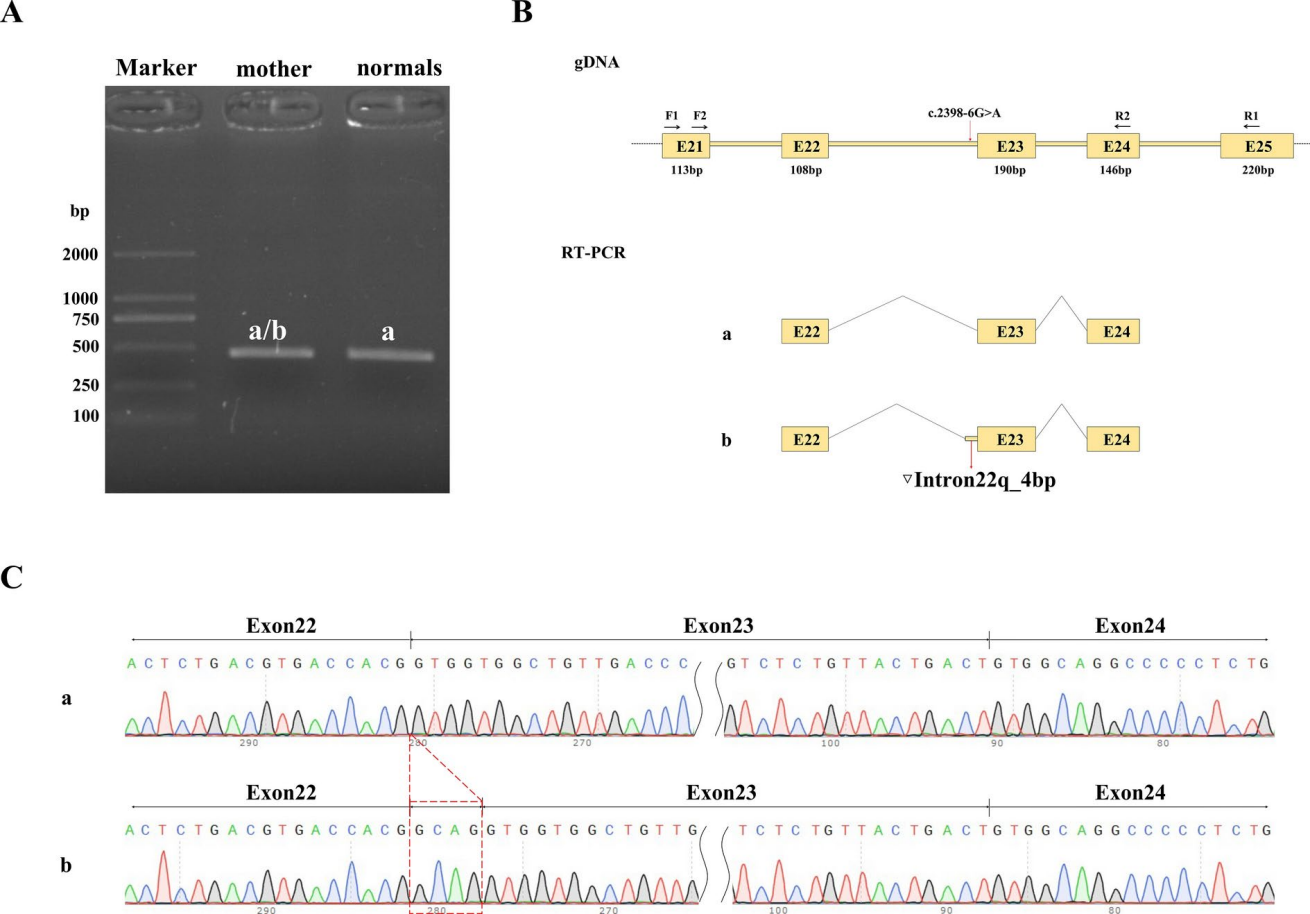
In vivo functional analysis of splicing defects induced by *CDH23* mutations. **a** Agarose gel electrophoresis of RT-PCR amplification products derived from the mother and a normal control, illustrating the differential splicing patterns. **b** Schematic overview of the RT-PCR primer design and the resultant splicing pattern. The red arrow indicates the mutation site that potentially disrupts normal splicing. **c** Sanger sequencing of RT-PCR products corresponding to shear bands a and b, and the red dashed box shows the retention of 4 nucleotides: GCAG between exon 22 and exon 23

In vivo RNA analysis further elucidated the impact of the heterozygous *CDH23* c.2398-6G > A variant carried by the mother. RT-PCR analysis revealed the retention of four nucleotides (GCAG) between exon 22 and exon 23, which is not present in the normal control (Fig. 3a). This splice variant disrupts normal mRNA splicing, leading to an abnormal splice product with the insertion of these nucleotides. The splice variant: c.2398-6G > A affected the normal mRNA splicing, produce an abnormal splice product with the insertion of these nucleotides. Consequently, the cDNA representation of the variant is c.2397_2398insGCAG (p. Val800Alafs*6), leading to a frameshift mutation and premature termination codon (PTC) within exon 23 (Fig. 3b, c). This mutation likely produces a length of 804 truncated protein. According to the American College of Medical Genetics and Genomics (ACMG) (Richards et al. 2015) guidelines for interpretation of sequence variants, this variant is classified as likely pathogenic (PS3 + PM3 + PM2 + PP3).

### Effect of mutations on protein structure

**Fig. 4:**
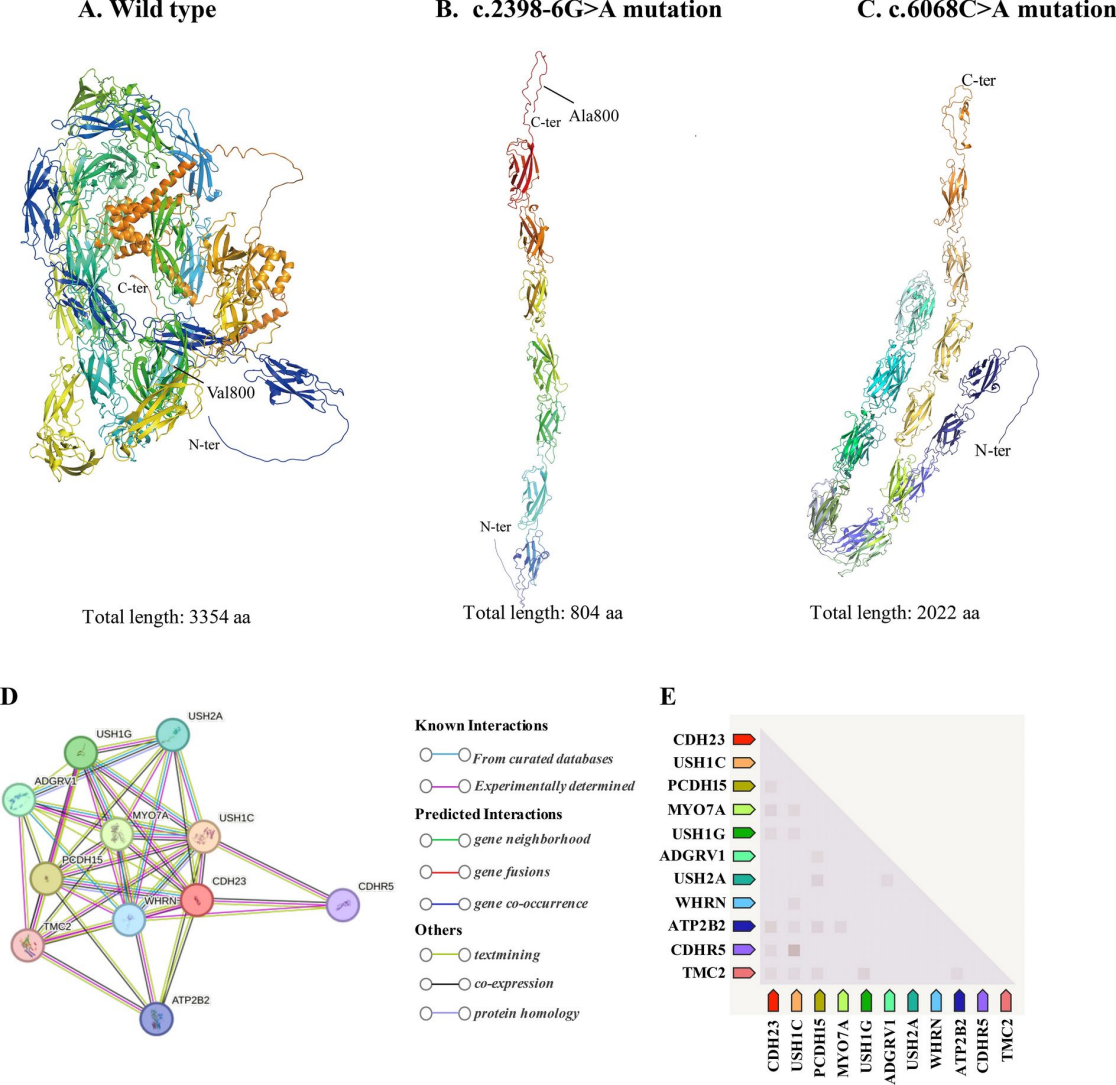
Structural modeling of *CDH23* protein to assess the impact of mutations. **a** Predicted three-dimensional structure of the wild-type *CDH23* protein, serving as the reference model. **b** Structural consequences of the c.2398-6G > A mutation on the *CDH23* protein, indicating potential alterations in protein folding or function. **c** Structural impact of the c.6068C > A mutation on the *CDH23* protein, illustrating how this mutation may compromise the protein’s integrity or interaction with other molecular components

**Fig. 5:**
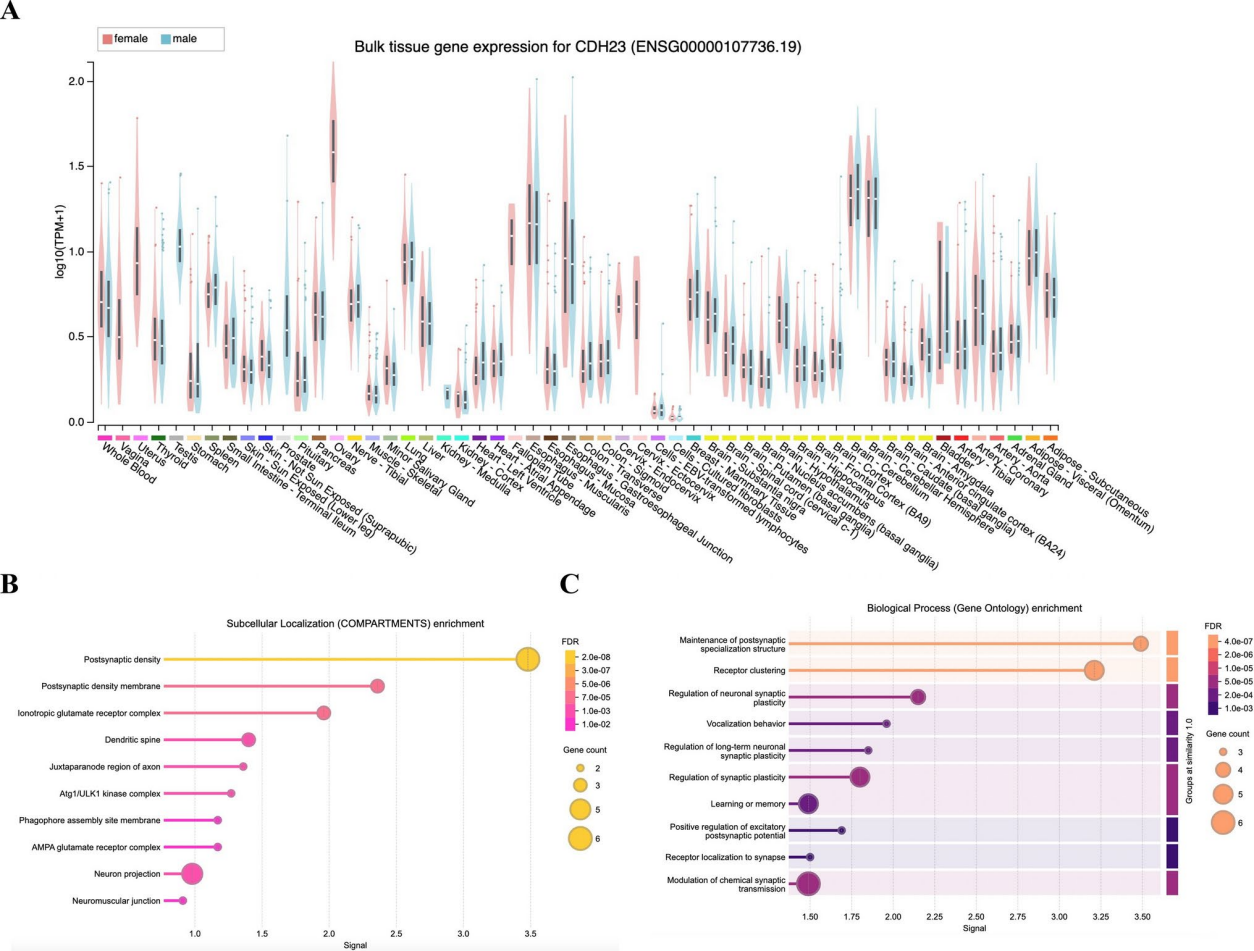
Bioinformatics analysis of the *CDH23* gene expression, localization and networks. **a** GTEx data demonstrate that *CDH23* is expressed at an appreciable level in adult human tissue represented (https://gtexportal.org/home/gene/CDH23). **bc** Subcellular localization and biological pathway were obtained through String data (https://string-db.org/cgi/network)

In order to analyze the effect of the two novel variants respectively, NM_022124.6: c.2398-6G > A, (p. Val800Alafs*6) and c.6068C > A, (p. Ser2023Ter) on protein structures *CDH23*, we used AlphaFold web server (https://golgi.sandbox.google.com/about) to predict changes in *CDH23* protein structures when there were mutations in the sequence. The NM_022124.6: c.2398-6G > A variant in the *CDH23* gene could induce a frameshift mutation, resulting in a truncated *CDH23* protein comprising 804 of the 3354 amino acids of the mature protein. This frameshift mutation is likely to significantly alter the three-dimensional protein structure of *CDH23* (Fig. 4b). The NM_022124.6: c.6068C > A, (p. Ser2023Ter) variant in *CDH23* gene will change the three-dimensional structure to a truncated *CDH23* protein that consist 2022 of the 3354 amino acids of the mature protein (Fig. 4c). This structural change would alter the conformation of the *CDH23* protein and affect the protein stability and binding facility. The PPI network analysis of interacting genes with *CDH23* was produced by STRING. As shown in Fig. 4d, e and Table 4, the PPI network consisted of 10 genes with interesting functional roles. Meanwhile, *CDH23* expressed widely around the different tissue and localized mainly on postsynaptic region (Fig. 5a, b). Pathway analysis suggested that *CDH23* and its interacting proteins are closely related to synaptic plasticity (Fig. 5c).

**Table 4:** Names and functions of predicted CDH23-interacting proteins

Interacting proteins	Function of proteins	Co-expr.score
USH1C	Harmonin; USH1 protein network component harmonin	0.999
PCDH15	Protocadherin-15; Calcium-dependent cell-adhesion protein. Essential for maintenance of normal retinal and cochlear function	0.999
MYO7A	Unconventional myosin-VIIa; Myosins are actin-based motor molecules with ATPase activity. Unconventional myosins serve in intracellular movements	0.998
USH1G	Usher syndrome type-1G protein; Anchoring/scaffolding protein that is a part of the functional network formed by USH1C, USH1G, CDH23 and MYO7A that mediates mechanotransduction in cochlear hair cells, which are required for normal hearing	0.992
ADGRV1	Adhesion G-protein coupled receptor V1; G-protein coupled receptor which has an essential role in the development of hearing and vision. Couples to G-alpha(i)-proteins, GNAI1/2/3, G-alpha(q)-proteins, GNAQ, as well as G-alpha(s)-proteins, GNAS, inhibiting adenylate cyclase (AC) activity and cAMP production	0.970
USH2A	Usherin; Involved in hearing and vision as member of the USH2 complex. In the inner ear, required for the maintenance of the hair bundle ankle formation, which connects growing stereocilia in developing cochlear hair cells	0.947
WHRN	Whirlin; Involved in hearing and vision as member of the USH2 complex. Necessary for elongation and maintenance of inner and outer hair cell stereocilia in the organ of Corti in the inner ear. Involved in the maintenance of the hair bundle ankle region, which connects stereocilia in cochlear hair cells of the inner ear	0.946
ATP2B2	Plasma membrane calcium-transporting ATPase 2; This magnesium-dependent enzyme catalyzes the hydrolysis of ATP coupled with the transport of calcium out of the cell	0.900
CDHR5	Cadherin-related family member 5; CDHR5 interacts with microvillus cytoplasmic proteins, forming the intermicrovillar adhesion complex (IMAC), which is crucial for the differentiation of microvilli and the epithelial brush border	0.881
TMC2	Transmembrane channel-like protein 2; Probable ion channel required for the normal function of cochlear hair cells. Component of the hair cell's mechanotransduction machinery. Involved in mechanosensitive responses of the hair cells	0.875

Co-expr.score: Co-expression score

## Discussion

Hearing loss adversely impacts language development, acquisition, and the social and cognitive maturation of affected children (Yang et al. 2023). In this study, the girl presented with hearing loss and regression of speech. Comprehensive medical histories and physical examinations, with a particular emphasis on audiological assessments, were conducted to exclude syndromic forms of hearing loss. Trio-based WES analysis was performed on the family, leading to the identification of two novel variants in the *CDH23* gene.

DFNB12 arises from mutations in the *CDH23* gene, located on chromosome 10q22, either in a homozygous or compound heterozygous state (Chaib et al. 1996). The non-syndromic form of DFNB12 hearing loss is usually linked to missense mutations in *CDH23*, which are believed to act as hypomorphic alleles. These alleles possess enough residual activity to maintain retinal and vestibular functions but fall short in supporting proper auditory function in the cochlea. A significant amount of research shows that USH1D is caused by homozygous nonsense, frameshift, and splice site mutations (primarily in canonical splice sites (Aparisi et al. 2013)), as well as certain missense mutations, or a combination of these mutations in a compound heterozygous configuration. (Bork et al. 2001a, 2002; Bolz and Roux 2011; Valero et al. 2019). Research has thoroughly established that USH1D is linked to homozygous nonsense, frameshift, and splice site mutations, especially those found at canonical splice sites, along with missense mutations in the *CDH23* gene. Furthermore, a combination of these USH1D alleles in individuals with compound heterozygosity may also contribute to the development of the condition.

According to the human SNP pathogenic data on the Malacards website (https://www.malacards.org/), at least 15% of pathogenic mutations in the human SNP database are located within introns, with the majority of these intronic mutations occurring near exon–intron boundaries, primarily affecting mRNA splicing. Most pathogenic variants that disrupt splicing alter the canonical splice sites, but other variants located at the flanking regions, known as noncanonical splice sites (NCSS), are increasingly recognized as causes of aberrant splicing events. Intronic NCSS variants at splice donor sites are predominantly situated at positions + 3 to + 6 downstream from exons, while intronic NCSS variants at splice acceptor sites are located at positions − 14 to − 3 upstream of exons (Sangermano et al. 2018). These are often classified as variants of unknown VUS (Shaikh et al. 2018). The Human Gene Mutation Database indicated that NCSS and nonsense mutations in *CDH23* could cause both USH1D and DFNB12 deafness (Valero et al. 2019). In this study, we identified two novel variants include NCSS and nonsense mutation, which led to the accurate diagnosis of the proband as DFNB12. It has been reported that two family members with compound heterozygous mutations in *CDH23* resulting in DFNB12 have passed a newborn hearing screening, suggesting that their hearing loss is not congenital or was very mild at birth (Schultz et al. 2011). Similarly, the girl in our study also passed her newborn hearing screening. Wagatsuma et al. (2007) reported five unrelated Japanese families with DFNB12, all exhibiting a consistent phenotype characterized by moderate to profound high-frequency progressive sensorineural hearing loss. The average hearing loss among these patients was 84.0 dB HL, which closely aligns with our study's findings, where the proband's average hearing loss in both ears was approximately 85 dB HL.

*CDH23* plays a crucial role in the proper organization of the static cilia bundle, and mutations in the *CDH23* gene disrupting stereocilia structure. These disruptions impair the conversion of sound waves into electrical signals in hair cells, leading to noise-induced hearing loss and age-related hearing loss (Noben-Trauth et al. 2003). Yang et al. (2023) demonstrated that loss of *CDH23* resulted in defective purine metabolism, subsequently insufficiency of ATP, which is of great importance for the normal function of hair cells. Patients with *CDH23* mutations displayed a wide range of hearing loss and RP phenotypes, varying in severity, age at onset, type, and the presence or absence of vestibular areflexia (Astuto et al. 2002). In our study, the girl presented with bilateral profound sensorineural hearing loss but did not exhibit any additional phenotypes.

It is noteworthy that homozygous c.109G > A variants in GJB2 were identified in each member of this family including the patient’s unaffected parents and sister. Mutations in the gap-junction protein gene GJB2 (OMIM: 121,011) were the most common cause for autosomal recessive hearing impairment (HI) (Kelley et al. 1998). The *GJB2* c.109G > A/p.Val37Ile has been increasingly studied recently due to its high prevalence in East Asians (Li et al. 2012; Kim et al. 2013), with an estimated 5 millions East Asians being homozygous for p.V37I variant (Chai et al. 2015). Initially, this variant was classified as a benign polymorphism for its high prevalence in normal hearing individuals (Kelley et al. 1998). However, recent studies have demonstrated that homozygous p.V37I can result in mild to moderate HI, albeit with reduced penetrance (Pollak et al. 2007; Gallant et al. 2013). Chai et al. (Chai et al. 2015) reported that homozygous p.V37I exhibits a relatively low penetrance (17%), with the majority of individuals carrying this variant avoiding significant hearing impairment until early adulthood. A large population screening in Shanghai revealed that biallelic p.V37I variant in *GJB2* led to slow but steadily progressive hearing loss, with increasing incidence with age. Most individuals with the biallelic p.V37I likely develop significant hearing loss in adulthood. Notably, none of the individuals with homozygous c.109G > A variants in the 7- and 15-year-old groups exhibited moderate or severe (≥ 50 dB HL) hearing loss (Chen et al. 2022b). In our study, the two novel pathogenic variants in the *CDH23* gene, rather than the homozygous c.109G > A variants, are responsible for the profound hearing loss (85 dB HL) observed in the 8-year-old girl. Patients with *CDH23* mutations have been predicted to achieve satisfactory auditory and speech outcomes following cochlear implantation (Yoshimura et al. 2020; Chen et al. 2022a). It is anticipated that a cochlear implant will significantly enhance the hearing of the girl identified in our research. Therefore, identifying the genetic etiology of hearing loss in affected individuals is advantageous, as it can enhance prognostic accuracy in clinical settings. In conclusion, our analyses revealed novel compound heterozygous mutations in *CDH23* associated with autosomal recessive NSHL, thereby expanding the mutational landscape of *CDH23*-related hearing loss and increasing knowledge about the *CDH23* splice site variants.

## Data Availability

The authors confirm that the data supporting the findings of this study are available from the corresponding author on request.
